# Mobilising vitamin D from adipose tissue: The potential impact of exercise

**DOI:** 10.1111/nbu.12369

**Published:** 2019-02-03

**Authors:** A. Hengist, O. Perkin, J. T. Gonzalez, J. A. Betts, M. Hewison, K. N. Manolopoulos, K. S. Jones, A. Koulman, D. Thompson

**Affiliations:** ^1^ Department for Health University of Bath Bath UK; ^2^ Institute of Metabolism and Systems Research University of Birmingham Birmingham UK; ^3^ NIHR BRC Nutritional Biomarker Laboratory University of Cambridge Cambridge UK

**Keywords:** 25(OH)D, 25‐hydroxyvitamin D, adipose, exercise, physical activity, vitamin D

## Abstract

Vitamin D is lipophilic and accumulates substantially in adipose tissue. Even without supplementation, the amount of vitamin D in the adipose of a typical adult is equivalent to several months of the daily reference nutrient intake (RNI). Paradoxically, despite the large amounts of vitamin D located in adipose tissue, individuals with obesity are often vitamin D deficient according to consensus measures of vitamin D status (serum 25‐hydroxyvitamin D concentrations). Thus, it appears that vitamin D can become ‘trapped’ in adipose tissue, potentially due to insufficient lipolytic stimulation and/or due to tissue dysfunction/adaptation resulting from adipose expansion. Emerging evidence suggests that exercise may mobilise vitamin D from adipose (even in the absence of weight loss). If exercise helps to mobilise vitamin D from adipose tissue, then this could have important ramifications for practitioners and policymakers regarding the management of low circulating levels of vitamin D, as well as chronically low levels of physical activity, obesity and associated health conditions. This perspective led us to design a study to examine the impact of exercise on vitamin D status, vitamin D turnover and adipose tissue vitamin D content (the *VitaDEx* project). The *VitaDEx* project will determine whether increasing physical activity (via exercise) represents a potentially useful strategy to mobilise vitamin D from adipose tissue.

## Introduction

Vitamin D has effects far beyond its classical actions on calcium homeostasis and bone metabolism, and vitamin D insufficiency is thought to affect many physiological systems and a wide array of human health outcomes (Dobnig *et al*. 2008; Ginde *et al*. 2009; Semba *et al*. 2010). Vitamin D status is conventionally determined by measuring concentrations of the main circulating form of vitamin D, 25‐hydroxyvitamin D [25(OH)D]. Approximately 30–40% of the UK population have circulating 25(OH)D <25 nmol/l in winter (SACN 2016), and the correction of low systemic concentrations of 25(OH)D is a recognised public health priority (Palacios & Gonzalez 2014; Cashman *et al*. 2016).

## Vitamin D: Overview

The two main forms of vitamin D are vitamin D_2_ (ergocalciferol) and vitamin D_3_ (cholecalciferol) (Askew *et al*. 1930; Windaus *et al*. 1936). Previtamin D_3_ is synthesised by the skin when solar ultraviolet (UV) radiation (wavelength 290–320 nm) penetrates the skin and is absorbed by 7‐dehydrocholesterol (Maclaughlin *et al*. 1982), before spontaneously and rapidly isomerising to vitamin D_3_. Vitamin D_3_ is metabolised in the liver to 25(OH)D – the main circulating form of vitamin D (Blunt *et al*. 1968; Blunt & Deluca 1969). Vitamin D_2_ and vitamin D_3_ can be obtained from the diet and from supplements (Holick 2007). Vitamin D_2_ is synthesised by UV irradiation of ergosterol and intake is primarily through consumption of UV‐irradiated mushrooms or supplements. In contrast, vitamin D_3_ is more widely distributed in foods and is more commonly used in supplements and fortified foods than vitamin D_2_. One reason for using serum 25(OH)D as a measure of vitamin D status is that it has a half‐life of ~2–3 weeks and, therefore, quantification of this metabolite is not influenced by transient changes in dietary vitamin D or acute sun exposure to the same extent as other vitamin D metabolites (Jones *et al*. 2015). The methods of assessing 25(OH)D are reviewed by Le Goff *et al*. (2015). In this review, reference to ‘vitamin D’ denotes either vitamin D_3_ (cholecalciferol) or 25(OH)D.

The main metabolically active form of vitamin D *in vivo* is the seco‐steroid 1,25‐dihydroxyvitamin D [1,25(OH)_2_D], also referred to as calcitriol. It is 1,25(OH)_2_D that is generally considered responsible for the physiological functions of vitamin D. The production of 1,25(OH)_2_D takes place predominantly in the kidney, and its action is mediated through binding to a vitamin D receptor (VDR), which is usually located in the nuclei of target cells, and which regulates target gene expression when bound with 1,25(OH)_2_D (Haussler *et al*. 2013). Study of VDR‐ablated mice has demonstrated numerous functions of vitamin D (Bouillon *et al*. 2013).

Vitamin D binding protein (DBP) in serum binds to different vitamin D metabolites with different affinity (Daiger *et al*. 1975). Vitamin D is bound to DBP for transport to relevant tissues and to regulate bioavailability (Safadi *et al*. 1999). Normally ~85% of circulating 1,25(OH)_2_D is bound to DBP, with ~0.4% being free circulating (Bikle *et al*. 1984, 1985), and ~88% of circulating 25(OH)D is bound to DBP with ~0.04% free (Bikle *et al*. 1986). Vitamin D can also be bound to albumin and chylomicrons of lipoproteins at lower affinity (Haddad *et al*. 1993). Although the majority of circulating vitamin D metabolites are bound to DBP or albumin, there is currently considerable debate about whether bound or unbound forms of vitamin D are biologically active (Bikle *et al*. 2017), with some tissues requiring uptake of DBP‐bound vitamin D and others appearing to access free or unbound vitamin D (Chun *et al*. 2014).

Potentially meaningful quantities of vitamin D have been observed in the skin, liver, skeletal muscle and adipose tissue of humans (Mawer *et al*. 1972), with evidence that extra‐renal tissues (such as the placenta) are capable of metabolising vitamin D (Weisman *et al*. 1979; Adams & Hewison 2012). The present review will focus on the role of adipose tissue, exploring the accumulation of vitamin D in this sizeable depot, whilst also exploring the potential mechanisms underlying the mobilisation of vitamin D from adipose within the context of well‐established physiological concepts.

## Vitamin D accumulation in adipose

Vitamin D is lipophilic and early studies used radioactive isotopes to demonstrate its accumulation in adipose tissue (Rosenstreich *et al*. 1971; Mawer *et al*. 1972). Supplementing with 20 000 international units (IU) of vitamin D_3_ per week for 3–5 years leads to a substantial increase in vitamin D_3_ content in subcutaneous abdominal adipose tissue, approximately sixfold greater than placebo (Didriksen *et al*. 2015). The amount of 25(OH)D present in adipose explants remained correlated with serum 25(OH)D concentrations 1 year after supplementation had ceased (Martinaityte *et al*. 2017). Although it is worth noting that values for serum 25(OH)D and adipose tissue vitamin D_3_ measured in this study varied considerably between participants, which may be explained by the fourfold variation in body fat mass across the study population. There is a positive linear correlation between vitamin D_3_ concentrations in subcutaneous abdominal adipose tissue and serum (Blum *et al*. 2008b). High concentrations of vitamin D_3_ have been observed in adipose tissue from various sites (perirenal, pericardial, axillary and cervical) taken from humans who were not known to be supplementing with vitamin D (Lawson *et al*. 1986). Vitamin D storage was highly variable between adipose depots (Lawson *et al*. 1986), which may be driven by depot‐specific differences in adipose tissue blood flow or metabolism; differences which have been identified when comparing subcutaneous abdominal adipose and gluteofemoral adipose (Manolopoulos *et al*. 2010). Furthermore, the relative amount of vitamin D (*i.e*. per gram of subcutaneous abdominal adipose tissue) is highly variable between individuals (Blum *et al*. 2008b; Pramyothin *et al*. 2011). It has recently been suggested that vitamin D accumulates in greater concentrations in omental adipose than subcutaneous abdominal adipose (Carrelli *et al*. 2017). However, given that the mass of subcutaneous adipose tissue is fourfold to sixfold greater than that of visceral adipose tissue (Ross *et al*. 2000; Merlotti *et al*. 2017), the absolute capacity and accumulation of vitamin D in subcutaneous adipose depots is likely to be quantitatively more important. Nonetheless, further research is needed to understand both the distribution and inter‐/intra‐individual variability of vitamin D accumulation across different adipose depots.

**Figure 1:**
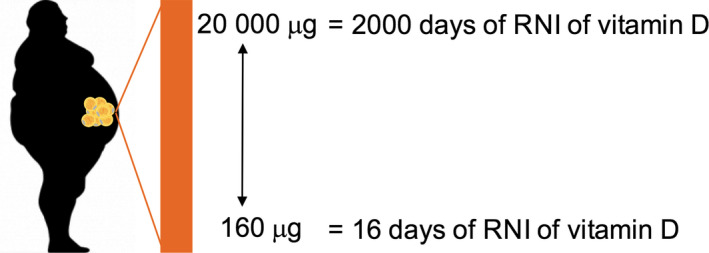
The amount of vitamin D present in adipose tissue is likely to be quantitatively important. When extrapolated, the amounts of vitamin D present in the adipose tissue of an individual weighing 100 kg and with a body fat percentage of 40% may equate to up to 2000 days of the daily recommended nutrient intake (RNI) of vitamin D (10 μg) from dietary sources or supplementation. [Colour figure can be viewed at wileyonlinelibrary.com]

Whilst adipose can accumulate both vitamin D_3_ and 25(OH)D, the limited data available suggest that the concentrations of vitamin D_3_ are much greater (Piccolo *et al*. 2013; Didriksen *et al*. 2015). Published values for the amount of vitamin D_3_ present in subcutaneous adipose tissue vary substantially, ranging from ~4 to ~500 ng/g, suggesting large individual variability and dependency on supplementation status (Didriksen *et al*. 2015). For an individual weighing 100 kg, with 40% body fat, this may equate to 160–20 000 μg vitamin D_3_, which is the equivalent to anywhere between 16 and 2000 days of the daily reference nutrient intake (RNI) of total dietary vitamin D (10 μg) for the UK population (Fig. 1). The median value for vitamin D_3_ in adipose of *non‐supplementing* humans with overweight or obesity is 32 ng/g (Didriksen *et al*. 2015), which equates to 128 days of the RNI. Thus, adipose tissue has the potential to accumulate a substantial amount of vitamin D, especially when adipose mass is expanded (*i.e*. in overweight and obesity).

## Obesity and vitamin D status

Individuals with obesity have lower circulating 25(OH)D concentrations than individuals without obesity (Compston *et al*. 1981; Bell *et al*. 1985; Liel *et al*. 1988; Vimaleswaran *et al*. 2013; Pereira‐Santos *et al*. 2015; Walsh *et al*. 2016). The mechanisms explaining this have been debated. One suggestion is that reduced sunlight exposure is a behavioural tendency in individuals with obesity (Compston *et al*. 1981), but there is limited empirical evidence to support this. Wortsman *et al*. (2000) found that increases in serum 25(OH)D, induced by UV exposure, were attenuated in individuals with obesity, hypothesising that vitamin D is less bioavailable because it is sequestered in the expanded adipose tissue compartment. In support of this suggestion, in individuals supplemented with 700 IU/day of vitamin D for a year, the increase in serum 25(OH)D was inversely related to body mass index (BMI) (Blum *et al*. 2008a). Another study assessed serum 25(OH)D responses across 90 days with ingestion of a single oral bolus of 300 000 IU of vitamin D_3_ and showed that the increase in serum 25(OH)D concentration was blunted in individuals with overweight or obesity compared with lean controls (Camozzi *et al*. 2016). Lower vitamin D status has sometimes been attributed to volumetric dilution, with regression analysis revealing body mass as the strongest univariate predictor of vitamin D status (Drincic *et al*. 2012). If volumetric dilution was driving this observation, then lower vitamin D status may also be associated with expansion of muscle (and total) mass. However, when comparing winter levels of serum 25(OH)D in a range of non‐supplementing athletes (rugby league players), significantly *greater* mean serum 25(OH)D was observed compared with non‐supplementing, non‐athlete controls despite markedly greater body mass (reflective of large muscle mass) (Close *et al*. 2013). Furthermore, in university athletes, fat mass was a significant predictor of serum 25(OH)D concentrations but fat‐free mass was not (Heller *et al*. 2015). Collectively, these findings indicate that volumetric dilution *per se* does not explain the negative associations between mass and vitamin D status. This makes sense when considering the lipophilic nature of vitamin D. Indeed, it seems likely that the detrimental effect of obesity on vitamin D status is predominantly due to specific expansion of adipose mass rather than expansion of total mass or volume.

Early studies postulated that increased circulating 1,25(OH)_2_D with obesity reduces serum 25(OH)D by reducing hepatic synthesis of 25(OH)D via negative feedback (Bell *et al*. 1984, 1985). However, there is a weak negative relationship between fat mass and circulating 1,25(OH)_2_D (Parikh *et al*. 2004), calling into question the existence of a negative feedback mechanism by which production and accumulation of excess 1,25(OH)_2_D would reduce 25(OH)D synthesis. Alternatively, low circulating vitamin D in individuals with obesity may be attributable to increased metabolic clearance and enhanced uptake of vitamin D by adipose tissue (Liel *et al*. 1988). An upregulation of enzymes responsible for metabolising 25(OH)D to 1,25(OH)_2_D is unlikely to contribute to decreased serum 25(OH)D because of the significantly lower circulating levels of 1,25(OH)_2_D relative to 25(OH)D (~1:400) (Lips 2007). Wamberg *et al*. (2013) analysed the expression of a number of vitamin D hydroxylases in visceral and subcutaneous abdominal adipose tissue of lean women and women with obesity. There was no difference in CYP24A1 expression (a major enzyme responsible for catabolising vitamin D) between lean individuals and individuals with obesity. Similar results have since been replicated in a cohort of individuals with obesity (Di Nisio *et al*. 2017). Together, these studies suggest that CYP2J2‐25‐hydroxylation and 1α‐hydroxylation are impaired, rather than upregulated, in the adipose tissue of individuals with obesity, so do not support the idea that the decreased systemic 25(OH)D concentrations observed in obesity are caused by increased metabolism of vitamin D in adipose. Other recent investigations using stable isotopes indicate that there is no difference in the overall 25(OH)D half‐life with obesity (Walsh *et al*. 2016). Therefore, adipose does not seem to actively metabolise more vitamin D in individuals with obesity and there seems to be no effect of obesity on overall 25(OH)D metabolism and turnover. Instead, the most striking effect of obesity seems to be that adipose becomes a sink or reservoir for vitamin D.

## A role for vitamin D within adipose?

Adipose tissue comprises heterogeneous cells including adipocytes, various immune cells and preadipocytes. The cellular composition of adipose impacts on adipose inflammation (Bourlier *et al*. 2008) and the secretion of inflammatory mediators (Maury & Brichard 2010), which is particularly pertinent to individuals with obesity as it contributes to systemic low‐grade inflammation (Trim *et al*. 2018).

Treatment of human adipocytes *in vitro* with 1,25(OH)_2_D decreases secretion of interleukin 6 (Mutt *et al*. 2012), suggesting that 1,25(OH)_2_D inhibits phosphorylation of IкBα, which has an inhibitory effect on NF‐кB (Baeuerle & Baltimore 1988). NF‐кB induces transcription of pro‐inflammatory pathways (Baldwin 1996), so vitamin D is potentially anti‐inflammatory for adipose tissue if available to exert physiological actions. Vitamin D might also be important for the formation of new adipocytes, and this has been reviewed in detail elsewhere (Dix *et al*. 2018). Peroxisome proliferator‐activated receptor gamma (PPARγ) is thought to be the ‘master regulator’ of adipogenesis, with differentiation maintained by concomitant expression of PPARγ and CCAAT enhancer‐binding protein α (Rosen *et al*. 2002). Study of human preadipocytes suggests 1,25(OH)_2_D promotes adipogenesis (Nimitphong *et al*. 2012). Narvaez *et al*. (2013) found similar effects in human mesenchymal progenitor cells (hMPCs), where 1,25(OH)_2_D increased expression of adipogenic marker genes including fatty acid synthase, fatty acid‐binding proteins and PPARγ.

*In vitro* studies provide intriguing insights into potential functions of vitamin D within adipose tissue itself. Unfortunately, very little is known about the amount/distribution of vitamin D metabolites and the actions of 1,25(OH)_2_D in adipose tissue *in vivo*. Furthermore, it is important to note that simple measurement of vitamin D_3_ or 25(OH)D in adipose biopsy samples does not reveal the extent to which active 1,25(OH)_2_D is available for biological activity.

The potentially positive effects of vitamin D on adipose tissue physiology and function present a very interesting avenue for further research. However, since vitamin D is likely sequestered in lipid droplets of adipocytes, it might only be expected to exert physiological effects within adipose once mobilised into the cytosol and/or interstitium. It is an intriguing paradox that, in individuals with obesity, vitamin D seems to be ‘trapped’ in the lipid droplets of adipocytes in very close proximity to sites that may potentially benefit if only it could be made bioavailable.

## Physical activity/exercise and vitamin D status

**Figure 2:**
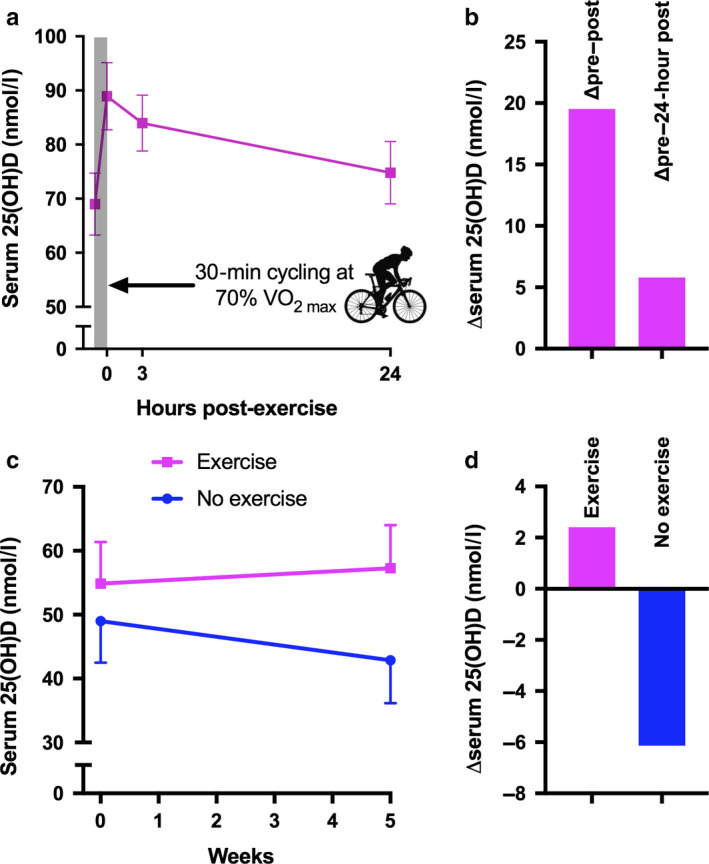
(a) The effect of 30 minutes of cycling exercise at 70% $\dot{V}O_{2}\;\max$ on serum 25(OH)D concentrations in humans [redrawn using data from Sun *et al*. (2017)]. Data shown are mean ± standard error of the mean (SEM) for men and women combined. (b) Mean change in serum 25(OH)D concentrations following 30 minutes of cycling exercise at 70% $\dot{V}O_{2}\;\max$. Left‐hand bar represents change from pre‐exercise to immediately post‐exercise, right‐hand bar represents change from pre‐exercise to 24 hours post‐exercise [redrawn using data from Sun *et al*. (2017)]. (c) The effect of 5 weeks of progressive cycling endurance exercise (30 minute bouts, 3 bouts/week, from 60% to 75% $\dot{V}O_{2}\;\mathrm{peak}$) during winter months on serum 25(OH)D [redrawn using data from Sun *et al*. (2018)]. Data shown are mean ±SEM. (d) Mean change in serum 25(OH)D concentrations following 5 weeks of progressive cycling endurance exercise [redrawn using data from Sun *et al*. (2018)]. [Colour figure can be viewed at wileyonlinelibrary.com]

Physical activity is a powerful stimulus for lipid mobilisation from adipose tissue (Thompson *et al*. 2012). It is therefore conceivable that vitamin D ‘trapped’ in adipocytes is mobilised (along with stored lipid) by physical activity. In support of this suggestion, association studies consistently report greater serum 25(OH)D concentrations in individuals who self‐report higher physical activity (Reinehr *et al*. 2007; Villareal *et al*. 2008; Chomistek *et al*. 2011; Mason *et al*. 2011; Gangloff *et al*. 2015; Chin *et al*. 2017). One study using objective measures of physical activity (hip‐based accelerometry) showed a trend for increased serum 25(OH)D at higher physical activity levels (Klenk *et al*. 2015). Such associations have often been attributed to confounding factors (*e.g*. active people spending more time outside and receiving additional sunlight exposure), but recent studies indicate that exercise may have a direct and causal effect on vitamin D status. Spoo *et al*. (2015) found a progressive increase in serum 25(OH)D in sled dogs performing prolonged endurance exercise across 8 days. Dietary vitamin D intake was controlled, there was no weight loss, and confounding from sunlight exposure was mitigated by the dogs’ thick fur coats (How *et al*. 1994). Furthermore, Sun *et al*. (2017) showed elevated serum 25(OH)D concentrations in lean humans in response to 30 minutes of cycling exercise at 70% $\dot{V}O_{2}\;\mathrm{peak}$ (Fig. 2a,b). The increase in circulating 25(OH)D was observed immediately post‐exercise and persisted for 24 hours. Interestingly, the strongest positive predictor of increased 25(OH)D (incremental area under the curve) across 24 hours was higher body fat percentage (with no such relationship with fat‐free mass). More recently, the same group reported that 5 weeks of progressive endurance cycling exercise (30 minute bouts, three bouts/week, 60–75% $\dot{V}O_{2}\;\mathrm{peak}$) during winter months prevented any reductions in serum 25(OH)D concentrations in a small group of elderly men (Sun *et al*. 2018) (Fig. 2c,d), independent from any changes in adipose tissue mass. Collectively, these recent studies indicate that exercise has a direct and causal effect on 25(OH)D concentrations – possibly through the mobilisation of adipose‐derived vitamin D and/or 25(OH)D.

## Lipolysis as a key mechanism of vitamin D mobilisation with exercise?

**Figure 3:**
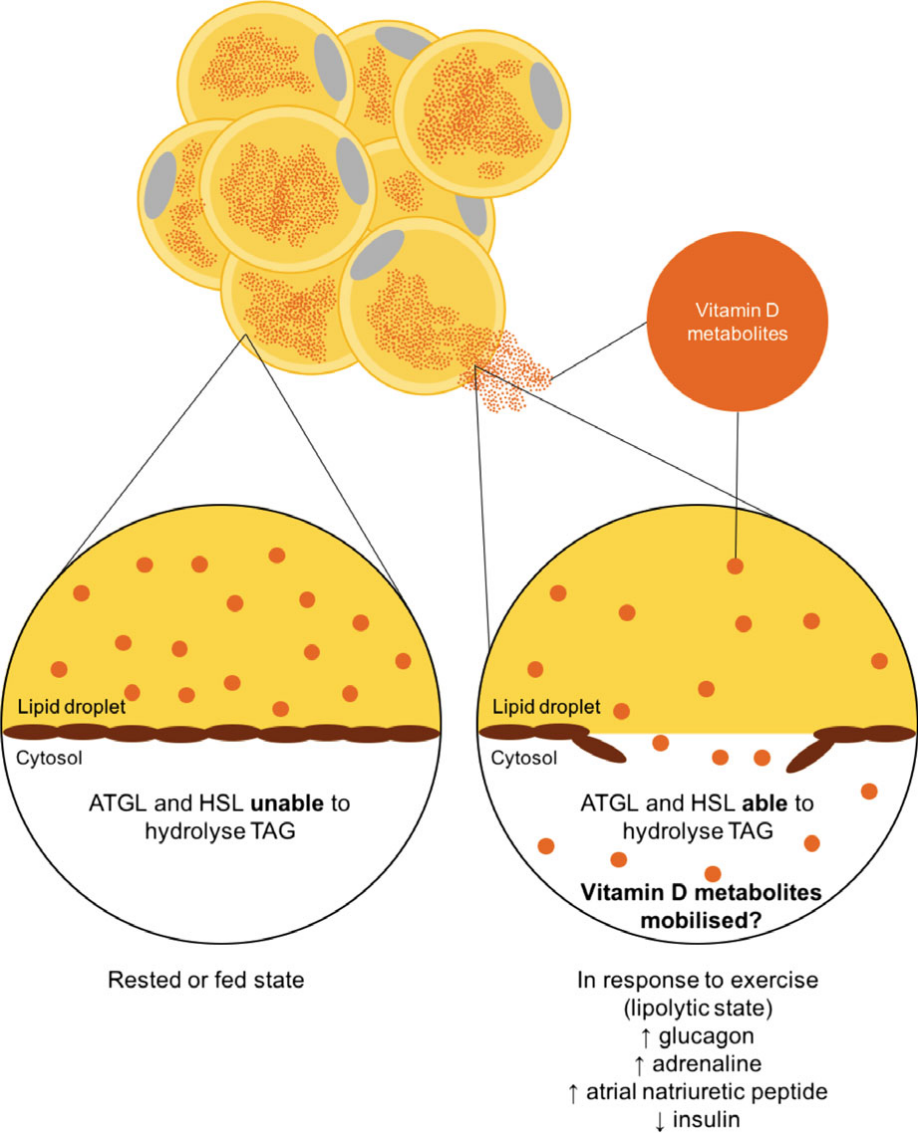
Vitamin D and the adipocyte. We hypothesise that in the rested or fed state triacylglycerol and vitamin D metabolites are trapped in the lipid droplet of adipocytes. When exposed to a lipolytic stimulus, triacylglycerol is liberated and this may coincide with mobilisation of vitamin D metabolites. ATGL, adipose triglyceride lipase; HSL, hormone‐sensitive lipase, TAG, triacylglycerol. [Colour figure can be viewed at wileyonlinelibrary.com]

During exercise, there is a rise in plasma glucagon, adrenaline and atrial natriuretic peptide (ANP) (Galbo *et al*. 1975; Bloom *et al*. 1976; Gyntelberg *et al*. 1977; Jezova *et al*. 1985; McMurray *et al*. 1987; Moro *et al*. 2007) and a decrease in plasma insulin (Hodgetts *et al*. 1991), concomitant with increased adipose tissue blood flow (Thompson *et al*. 2012). Glucagon, adrenaline and ANP are stimulatory lipolytic hormones (Arner *et al*. 1990; Perea *et al*. 1995; Moro *et al*. 2007) and suppression of insulin leads to a potent increase in lipolysis (Jensen *et al*. 1989). This leads to hydrolysis of triacylglycerol from the lipid droplet of adipocytes by the action of adipose triglyceride lipase (ATGL) (Jenkins *et al*. 2004; Villena *et al*. 2004; Zimmermann *et al*. 2004) and hormone‐sensitive lipase (HSL) (Vaughan *et al*. 1964). Exercise in the fasted or the fed state leads to an approximate twofold to threefold increase in adipose tissue lipolysis (Wolfe *et al*. 1990; Klein *et al*. 1994; Enevoldsen *et al*. 2004) and, when stored triacylglycerol is hydrolysed, vitamin D metabolites may also be released from the lipid droplet (Fig. 3).

The lipolytic response of adipose tissue to exercise is impaired in individuals with obesity. *Ex vivo* adipose tissue explants from individuals with obesity display reduced maximal lipolytic responses to adrenergic stimulation compared with adipose from individuals without obesity (Reynisdottir *et al*. 1994; Large *et al*. 1999; Hellstrom & Reynisdottir 2000), coinciding with decreased gene expression of ATGL and HSL (Large *et al*. 1999; Langin *et al*. 2005; Jocken *et al*. 2007; McQuaid *et al*. 2011). These findings are supported *in vivo*, as individuals with obesity display lower rates of lipolysis during exercise than lean controls (Stich *et al*. 2000; Mittendorfer *et al*. 2004). Whilst this may protect individuals with obesity from elevated circulating fatty acids (McQuaid *et al*. 2011), it may also contribute to sequestration of vitamin D. In support of this contention, adipose explants taken from individuals with obesity release less vitamin D when stimulated with lipolytic hormones than explants from lean controls (Di Nisio *et al*. 2017). In this study, mobilisation of vitamin D was proportional to both the lipolytic response (glycerol release) and protein expression of beta‐adrenergic receptors (Di Nisio *et al*. 2017); further supporting the notion that vitamin D mobilisation is inherently coupled to lipolysis.

Exercise may also target adipose tissue dysfunction in obesity. It is well established that an increase in physical activity chronically improves adipocyte function (Thompson *et al*. 2012). Exercise training increases sensitivity to various mediators such as insulin and adrenaline in overweight/obese adipose tissue (Thompson *et al*. 2012). It has also recently been shown that trained individuals exhibit greater protein content of lipolytic enzymes in subcutaneous abdominal adipose tissue (Bertholdt *et al*. 2018). Thus, in addition to an acute effect on vitamin D release associated with each exercise bout, regular exercise improves adipocyte function and the capacity to respond when stimulated, which may include the capacity to mobilise vitamin D in response to multiple stimuli (*e.g*. habitual physical activity, fasting and stress).

## The effect of exercise on vitamin D mobilisation: The VitaDEx project

The UK Biotechnology and Biological Sciences Research Council (BBSRC) recently supported the *VitaDEx* project (‘Mobilising Vitamin D sequestered in adipose tissue in humans with Exercise’; running from October 2018 to October 2021). In *VitaDEx*, we will examine the impact of increased exercise on whole‐body and adipose tissue vitamin D metabolism, along with the pathways involved in vitamin D mobilisation from adipose tissue. We will use a 10‐week randomised controlled trial in overweight men and women to determine the impact of exercise (versus control) on vitamin D status and metabolism. Multiple vitamin D metabolites including vitamin D_3_, 25(OH)D_3_, 25(OH)D_2_, active vitamin D [1,25‐dihydroxyvitamin D, 1,25(OH)_2_D_3_] and other metabolites such as 24,25‐dihydroxyvitamin D [24,25(OH)_2_D_3_] and 3‐epi‐25(OH)D will be measured in serum using liquid chromatography–mass spectrometry (LC‐MS/MS) (Jenkinson *et al*. 2016). Turnover of 25(OH)D will be assessed using an established stable isotope technique (with measurement by LC‐MS/MS) (Jones *et al*. 2016; Walsh *et al*. 2016). We will determine whether the change in serum 25(OH)D has a demonstrable effect on functional measures of bioavailability by culturing monocytes from a single donor with media supplemented with participant serum to assess the impact of different serum vitamin D concentrations on monocyte function. In addition, measures in subcutaneous abdominal adipose biopsies and fluxes across adipose tissue (arteriovenous differences) will be combined to understand the impact of exercise on the capacity to mobilise vitamin D from adipose tissue. The acquired tissue samples will enable us to characterise the biological pathways and mechanisms that are involved in vitamin D mobilisation. In addition to the intervention study, we will include a lean comparator group to understand the independent effects of adiposity and contextualise the effects of exercise on vitamin D mobilisation, status and metabolism.

Variability in UV‐induced vitamin D synthesis is a potential risk during intervention studies and, therefore, we will structure the experimental work to take place during the winter months when there is little vitamin D synthesis at the latitude where the trial will be conducted (Webb *et al*. 1988). This restriction in time for experimental work necessitates a pragmatic approach to study design; thus, a 10‐week exercise intervention has been chosen to increase the available window of time for participants to commence the trial. A 10‐week exercise intervention is also long enough to impact on key measures of adipose tissue function (Thompson *et al*. 2012). Furthermore, we anticipate that serum 25(OH)D will decline during 10 weeks in winter in non‐exercising control participants, such that protective effects of an exercise intervention will be apparent over this timeframe. Notably, all exercise undertaken by the exercise group will occur indoors. Participants in the exercise group will perform cardiovascular exercise training four times per week, predominantly in the form of treadmill walking and cycling. Exercise duration will be progressive, at an intensity that will be personalised to participants, predominantly corresponding to maximal rates of fat oxidation.

Another potential risk during exercise interventions is variability in energy balance and weight loss due to varying degrees of dietary compensation (Turner *et al*. 2010), so we will compensate for the increase in energy expenditure with food prescribed to fully offset the energy expended during exercise (thus maintaining energy balance). Energy expenditure will be monitored during exercise to enable us to replace the energy expended with foods containing no vitamin D, and we will verify the adequacy of this energy replacement by monitoring changes in body composition via dual X‐ray absorptiometry (DEXA). Dietary intake will be recorded with a 3‐day weighed diet record in the week prior to, and during the final week of, the intervention, along with a retrospective food frequency questionnaire at both time points to more specifically capture dietary sources of vitamin D habitually consumed.

## Conclusions

Vitamin D can accumulate in large amounts in adipose tissue where it may become sequestered. Preliminary evidence indicates that exercise may be a potential strategy to mobilise vitamin D from adipose tissue. We will examine this concept in a new research study (the *VitaDEx* project). This research will help us to understand the impact of exercise on vitamin D status and whether increasing physical activity represents a potentially useful strategy to mobilise vitamin D from adipose tissue. If exercise helps to mobilise vitamin D from adipose, then this could have important ramifications for practitioners and policymakers regarding the management of (i) low vitamin D status, (ii) obesity and associated conditions and (iii) low levels of physical activity. Current public health strategies typically approach vitamin D deficiency by increasing intake and/or synthesis of vitamin D (*e.g*. dietary supplementation or UV treatment). Notably, overweight/obesity reduces the impact of dietary supplementation with vitamin D on 25(OH)D concentrations (Arunabh *et al*. 2003; Snijder *et al*. 2005; Blum *et al*. 2008a; Beydoun *et al*. 2010) and the systemic 25(OH)D response to UV irradiation is significantly impaired (Wortsman *et al*. 2000). Thus, complementing conventional intake/synthesis strategies with techniques to mobilise endogenous vitamin D has the capacity to mutually enhance the overall effectiveness of interventions to improve vitamin D status.

## Conflict of interest

The authors have no conflict of interest to disclose.

## Author contribution

AH and DT conceptualised the work; AH and DT wrote the first draft; JTG provided initial intellectual insights; KSJ and MH provided further intellectual insights; all authors edited the manuscript and all authors agreed on the final version of the manuscript prior to submission.
